# The Multifaceted Roles of STAT3 Signaling in the Progression of Prostate Cancer

**DOI:** 10.3390/cancers6020829

**Published:** 2014-04-09

**Authors:** Jennifer L. Bishop, Daksh Thaper, Amina Zoubeidi

**Affiliations:** The Vancouver Prostate Centre, Department of Urologic Sciences, University of British Columbia, 2660 Oak Street, Vancouver British Columbia, V6H 3Z6, Canada; E-Mails: jbishop@prostatecentre.com (J.L.B.); dthaper@prostatecentre.com (D.T.)

**Keywords:** STAT3, androgen receptor, prostate cancer, castrate resistance, CSC, metastasis

## Abstract

The signal transducer and activator of transcription (STAT)3 governs essential functions of epithelial and hematopoietic cells that are often dysregulated in cancer. While the role for STAT3 in promoting the progression of many solid and hematopoietic malignancies is well established, this review will focus on the importance of STAT3 in prostate cancer progression to the incurable metastatic castration-resistant prostate cancer (mCRPC). Indeed, STAT3 integrates different signaling pathways involved in the reactivation of androgen receptor pathway, stem like cells and the epithelial to mesenchymal transition that drive progression to mCRPC. As equally important, STAT3 regulates interactions between tumor cells and the microenvironment as well as immune cell activation. This makes it a major factor in facilitating prostate cancer escape from detection of the immune response, promoting an immunosuppressive environment that allows growth and metastasis. Based on the multifaceted nature of STAT3 signaling in the progression to mCRPC, the promise of STAT3 as a therapeutic target to prevent prostate cancer progression and the variety of STAT3 inhibitors used in cancer therapies is discussed.

## 1. Introduction

**Figure 1:**
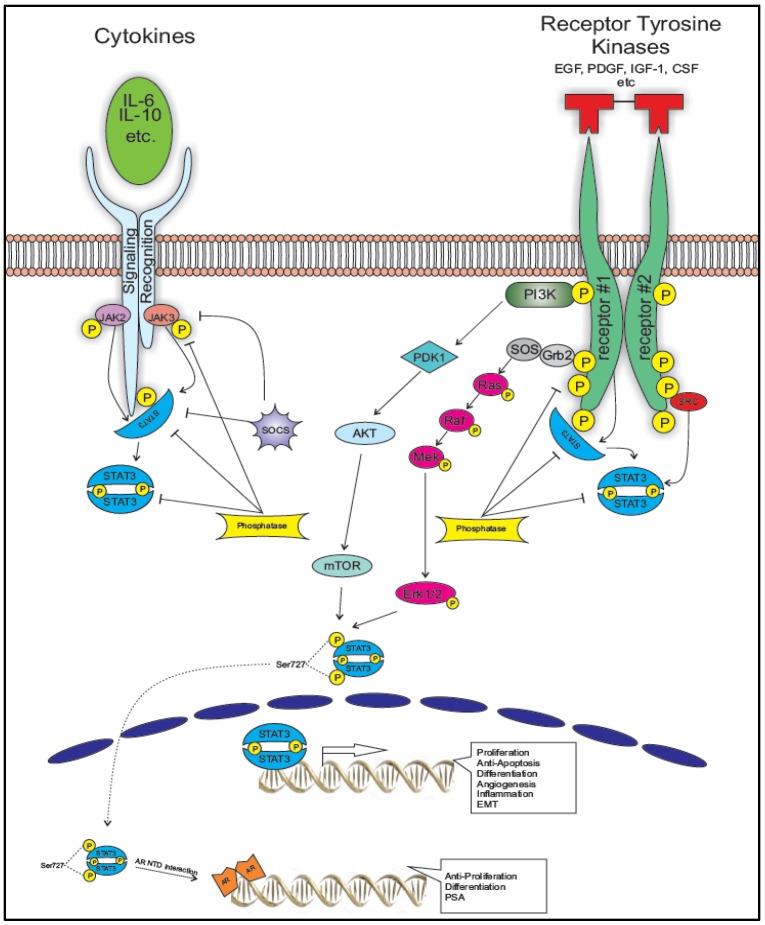
STAT3 integrates different signaling pathways involved in prostate cancer progression to metastatic disease. Binding of ligands to Cytokine Receptors and or Receptor Tyrosine Kinases recruits non-receptor Tyrosine kinases (JAK family and Src family) through their SH2 domains to the receptors. The same SH2 interaction also recruits STAT3 to the receptors and STAT3 gets phosphorylated on Tyrosine 705 by the non-receptor kinases leading to its dimerization, nuclear translocation and binding to DNA of target genes involved proliferation (CyclinD1, cMyc, Mcl1), Angiogenesis (Hif1α and VEGF), EMT (Twist, MMP2, 9, 7). In addition to this, the activity of mTOR and MAPK pathways phosphorylates STAT3 at Serine 727 which directly interacts with the NTD of the Androgen Receptor promoting its differentiation activity without increasing cell proliferation.

The signal transducer and activator of transcription (STAT)-3 plays an indispensable role in the progression of a wide variety of cancers. Activation of STAT3 downstream of cell surface receptors for cytokines and growth factors, by oncogenes or by chemical carcinogens in the microenvironment of many tumor types drives their transformation, survival, proliferation, invasion and dissemination. This is mediated by phosphorylation of latent cytoplasmic STAT3 on specific residues (Y705, Ser727) by a variety of tyrosine and serine kinases leading to its dimerization and nuclear translocation, where it acts as a transcription factor for a plethora of genes governing the malignant properties of the tumor cell. STAT3 is a critical mediator of differentiation, activation, migration and inflammatory capacity of immune cells and stromal cells that create the microenvironment supporting tumor cell growth (Figure 1). Importantly, there are strong feed forward mechanisms between the factors able to activate STAT3 and what genes STAT3 itself activates, intrinsically in tumor cells themselves as well as in the hematopoietic and stromal compartments, explaining perhaps the constitutive or at least hyperactivation of STAT3 in almost all cancers and highlighting its role as a bona fide oncogene. 

Overviews on the importance of STAT3 signaling and targeting it with potential therapies for cancer in general have been the subject of recent reviews [1]. However, the breadth and scope of STAT3 regulatory networks that drive the progression of prostate cancer (PCa) have not been addressed recently. In PCa, STAT3 plays a unique role; it acts as a key signaling conduit that allows activation of the androgen receptor (AR), a central driver of PCa cell survival and proliferation, by alternative mechanisms than through AR binding to its primary ligand, testosterone. These mechanisms include the IL-6 cytokine pathway as well as other oncogenic and molecular chaperone pathways known to drive the re-activation of the AR in castration-resistant prostate cancer (CRPC), including AKT/PI3K/PTEN, MAPK, EGFR and heat shock proteins (Hsps). As well, STAT3 controls PCa cell fate and interaction with the microenvironment; it plays roles in the maintenance of cancer stem cell (CSC) populations, the switch between epithelial to mesenchymal phenotypes that precede metastasis, tumor angiogenesis, as well as both tumor cell and stromal cell mediated immunosuppression. In this review, we will discuss the multifunctional role of STAT3 in PCa progression and drug resistance as well as its potential as a therapeutic target in this disease. 

## 2. STAT3 Signaling

STAT 3 belongs to the STAT family of highly conserved proteins that originally were identified in the acute phase response. Abbreviation for the Latin word for “immediately”, the STAT family of proteins is unique in that they control the fastest signaling pathway linking extracellular signals to a transcriptional response. STAT3 activation is most commonly associated with the binding of inflammatory cytokines or growth factors in the IL-6 [2] and IL-10 [3] family (G-CSF, CNTF, Oncostatin M, IL5-6, IL10-11, IL12, IL19-21 IL-22, IL-24, IL-26, IL-28) to their cognate receptors on the cell surface. Downstream of the activated cytokine receptor, STAT3 must be phosphorylated at two sites, tyrosine 705 and serine 727 for full activation [4]. Janus associated kinases (JAK) typically phosphorylate STAT3 on Y705, leading to its dimerization through the SH2 domain to opposing STAT3 monomers. However, Y705 can also be phosphorylated by other Receptor Tyrosine Kinases (RTK) directly including EGFR, VEGFR, PDGFR and IGFR [5], as well as non-receptor tyrosine kinases like Src-family kinases (Src, Lyn, Fyn, *etc*.) and Abl [6,7]. Phosphorylation of Serine 727 by serine/threonine kinases like p38MAPK [8], ERK [9], JNK [10], PKC [11] and mTOR [4] is required for optimal activity, allowing for Improtin α5 mediated nuclear transport of the STAT3 dimer [12]. Once in the nucleus, the DNA binding domain of each STAT3 monomer binds to an 8–10 nucleotide GAS (Gamma activated sequence) or ISRE (IFN-stimulated response element) to initiate gene transcription [13]. Interestingly, crystal structures of STAT3 bound to DNA indicate that dimers can exist without SH2 binding, indicating that non-phosphorylated STAT3 can dimerize to induce gene transcription, a feature which is unique to STAT3 in the STAT family [13]. 

Importantly, the specificity, duration and inhibition of STAT3 dependent gene transcription is tightly regulated. Because STAT3 signals downstream of so many extracellular cytokines and growth factors and it is activated by so many intracellular kinases, multiple levels of control dictate the specificity of which genes are transcribed in a STAT3 dependent manner, depending on the stimulus. For example, multiple co-activators like CBPp300 [14], APE1 [14] and NCOA [15] cooperate with STAT3 in specific conditions to control gene transcription. Moreover, the duration of STAT3 induced transcription is very tightly regulated [13]. Even under constitutive cytokine signaling, STAT3 phosphorylation peaks between 15–60 min. These phosphorylation events are tightly controlled by many different protein families, including protein tyrosine phosphatases, protein inhibitors of activated STATs (PIAS), proteasomal degradation and most importantly the family of suppressor of cytokine signaling proteins (SOCS) [16]. This regulation of phosphorylation events by SOCS family members combined with the number of STAT3 binding sequences present in the promoter region of a gene that dictate the strength of STAT3 mediated gene induction. This mechanism explains the contrasting effects of STAT3 mediated IL-6 *vs*. IL-10 gene transcription; the transient nature of IL-6 signaling is controlled by rapid SOCS3 inhibition of the gp130 subunit of the IL-6 receptor. This limits the duration that the STAT3 dimer is functional within the nucleus, allowing pro-inflammatory genes with multiple response elements to be transcribed. By contrast, the lack of a SOCS3 binding site in the IL-10 receptor prolongs STAT3 activation, allowing more time for STAT3 dimers to bind to anti-inflammatory genes that have only single STAT3 response elements [17]. In cases where unphosphorylated STAT3 dimers bind DNA, the altered dimer structure binds different response elements than the typical GAS and IRSE, allowing for transcription of varying genes [12,18]. For example, Yu *et al*. found that unphosphorylated-STAT3, through direct interaction with p65 of nuclear factor (NF)-κβ, serves as a suppressor and inhibits the ability of NF-κβ to induce the iNOS promoter in mesangial cells [19].

## 3. STAT3 in Oncogenic Signaling in PCa

Growth and maintenance of normal prostate epithelium and primary prostate cancer tumors is fueled by androgen activation of the AR present in prostate cells. This reliance on AR signaling makes androgen depravation therapy (ADT) an effective way to limit prostate cancer tumor growth; initially tumors respond well to anti-androgen therapy, dramatically shrinking in size and patients see a reduction in cancer symptoms. However, tumor recurrence within 3 years of ADT occurs in 80% of patients, and tumor regrowth combined with a rise in the AR dependent production of circulating prostate specific antigen (PSA), marks the progression to CRPC. Continued dependence on AR signaling is a hallmark of CRPC and there are multiple mechanisms by which the AR can be re-activated in castration conditions, including hyperactivation of oncogenic signaling pathways, intratumoral production of androgen, ligand independent AR activation due to AR mutation or splice variants and increased expression of molecular chaperones and the AR itself [20]. Any combination of these AR activating changes in CRPC tumors makes them hypersensitive to androgen and tumor growth resumes as in androgen dependent conditions [21,22]. While significant advancements in therapy have prolonged progression to CRPC, it still remains an incurable form of PCa.

Hyper-activation of multiple oncogenic signaling pathways drives progression of PCa to CRPC. For example, the most frequently deleted tumor suppressor gene in prostate cancer is phosphatase and tensin homolog gene (*PTEN*), which is a negative regulator of the PIK3/Akt survival pathway [23]. As a result of PTEN loss, constitutive activation of AKT and its downstream signaling targets can lead to activation of the AR in CRPC. Indeed there is areciprocal feedback loop whereby PI3K inhibition activates the AR and vice versa in the absence of PTEN [24]. Importantly, there is a close interplay between STAT3 and the PTEN/PI3k/AKT pathway. For example, in mice heterozygous for PTEN, development of adenocarcinoma is accelerated by the constitutive activation of STAT3. PTEN+/−STAT3C mice not only show increased phosphorylation of STAT3 but also AKT, which augment the growth rate of spontaneous tumors arising in these mice [25]. Highlighting the importance of feed forward mechanisms present in the tumor microenvironment mediated by a the synergy between STAT3 and PTEN loss is recent data from the Witte lab, showing that IL-6 and oncostatin M increase STAT3 signaling leading to the development of aggressive adenocarcinoma of the prostate in mouse models of PTEN loss or constitutive AKT expression [26]. Moreover, STAT3 is a direct transcriptional repressor of p53 and loss of p53 in combination with PTEN drives the progression of lethal CRPC [27].

In addition to the PTEN/PI3K/AKT pathway, STAT3 also functions up or downstream of a number of other oncogenes important for PCa progression. For example, STAT3 is required for the activation of proto-oncogenes that protect PCa cells from apoptosis, including Bcl-2 and Bcl-3 [28,29,30]. Interestingly, STAT3 is also a critical component downstream of the y box binding protein YB-1 in Her2 driven breast cancer as well as renal cancer. YB-1 protects breast cancer cells from apoptosis via activation of AKT/mTOR and phosphorylation of STAT3 at serine 727 [31] and in renal cancer YB1 stabilizes STAT3 and YB-1/STAT3 signaling mediate resistance to interferon a therapy [32]. These data have important implications for PCa, as YB-1 has been shown to be activated downstream of AKT and can activate the AR in CRPC [33]. It suggests that STAT3 may be a central signaling node downstream of a number of oncogenes, including both AKT and YB-1 that can lead to AR reactivation as well as support PCa cell survival. 

## 4. STAT3 as a Modulator of Androgen Receptor in PCa

External signals from cytokines and growth factors can trigger oncogenic signaling cascades that modulate AR activity. In particular, IL-6, the prototypical JAK/STAT3 signaling cytokine, can modulate activity and expression of the AR. It is no surprise therefore that increased IL-6 levels in PCa patients has been associated with decreased survival [34], increased metastasis [35] and related morbidity [36]. However, even though IL-6 seems to be associated with tumor progression, its relationship with the AR is convoluted and IL-6/STAT3 can have both pro and anti-proliferative effects on tumor cells as that depends on AR status of PCa cells well as activity/crosstalk from other pathways. For example short-term IL-6 treatment of the PCa cell line LNCaP activates STAT3 and AR downstream genes such as PSA, due to direct interaction between STAT3 residues 234–558 and the NTD of the Androgen Receptor [37]. Similarly, in pancreatic cancer cells treated with IL-6 there is increased phosphorylation of STAT3, which is required for AR transcriptional activity [38]. Interestingly, IL-6 mediated activation of STAT3 and AR is also associated with inhibition of cell growth and differentiation of LNCaP cells to a neuroendocrine phenotype [39]. By contrast, long-term treatment of cells with IL-6 greatly reduces AR expression [40] but at the same time, IL-6 loses its growth inhibitory effect [41] and the cell line shows more activity in the MAPK pathway rather than IL-6 induced STAT3 phosphorylation. Other studies have also determined that activation of STAT3 is an underlying mechanism for IL-6 induced growth inhibition, as IL-6 treated cells expressing dominant negative STAT3 show no signs of a growth inhibitory effect [39]. Independently, Lin *et al*. showed that IL-6 receptor can cooperate with ErbB2 (Her2/Neu) to activate the MAPK pathway with short-term IL-6 treatment and promote AR activity [42]. This interaction could also explain the previously mentioned increased proliferation caused with long-term IL-6 treatment’s activation of the MAPK pathway once AR expression is reduced. 

The link between IL-6 and STAT3 signaling to AR activity is further underscored by the importance of this signaling pathway in the development of anti-androgen resistance. Autocrine IL-6 signaling in PCa cells induces the constitutive activation of STAT3, enhancing recruitment of the AR to the PSA promoter and rendering these cells resistant to the new generation anti-androgen Enzalutamide, which can be reversed by treatment with a STAT3 inhibitor [43]. In addition, the feed forward loop that exists between IL-6, the NF-κβ pathway and STAT3 may also determine anti-androgen resistance. For example, both paracrine and autocrine IL-6 signaling activates NF-κβ in cancer cells, and there is substantial crosstalk between NF-κβ and STAT3, leading to their positive or negative regulation [44]. The significant overlap between tumor prosurvival and proliferative genes activated by STAT3 and NF-κβ, is not surprising based on data showing that STAT3 maintains the acetylation and nuclear retention of the NF-ΚΒ transcriptional subunit RelA in Du145 PCa cells as well as hematopoietic cells [45]. Importantly, like in autocrine IL-6 expressing cells, recent work has shown that overexpression of NF-κβ in LNCaP cells confers resistance to Enzalutamide and Enzalutamide treatment greatly increases NF-κβ expression. This resistance was associated with the NF-κβ dependent expression of AR splice variants which exhibit ligand independent activation of AR [46,47]. Although this study did not directly show the link between IL-6 and NF-κβ activation, others in PCa have shown these pathways are intrinsically linked and the importance of NF-κβ in PCa is widely accepted [48]. Taken together, these results suggest that while STAT3 activity may be associated with reduced PCa cell proliferation, STAT3 mediated signaling is an important component of the progression to CRPC and the development of anti-androgen resistance downstream of multiple pathways that are present in the PCa tumor environment. 

## 5. STAT3 as a Mediator or PCa Tumor Cell Phenotypic Plasticity: CSCs and EMT

While re-activation of the AR is undoubtedly the hallmark of progression to CRPC, it is also clear that the ability of PCa tumor cells to retain some degree of phenotypic plasticity, or the ability to change into a variety of aggressive tumor cell types, such as cancer stem cells or cells that have undergone epithelial to mesenchymal transition (EMT), is also a critical component of advanced disease. CSC theory postulates that transformed progenitor cells drive continued expansion of tumors with invasive and metastatic propensity [49]. CSCs have been found in many cancers, including prostate [50], and prostate cancer (PCa) patients with tumors harboring an embryonic stem cell signature have poor survival outcome with tumors that are significantly more likely to metastasize [51]. Because CSCs from prostate show resistance to chemotherapy and radiotherapy [52], it may be that the selective pressure of drugs used during CRPC treatment also cause PCa cells to acquire features of stem cells, leading to treatment resistance. Indeed, the frequency of PCa cells with stem markers increase in after castration in mice and CSC marker expression increases in basal PCa cells after ADT [53]. In addition to their intrinsic resistance to treatment and self-renewal properties, CSCs may promote treatment resistance through their ability to undergo EMT. EMT, a normally embryonic developmental program in which epithelial cells assume a mesenchymal phenotype during gastrulation and organogenesis, is activated by multiple signals in the tumor microenvironment, is regulated by many oncogenic signaling pathways and transcription factors, and is required for tumor metastasis [54]. Indeed, expression of mesenchymal markers is associated with invasiveness of PCa cell lines [55] and with high Gleason score and tumor metastasis in patients undergoing ADT [56]. 

Emerging evidence suggests that STAT3 plays critical roles in maintaining CSCs and promoting EMT in PCa as well as head and neck, hepatocellular carcinoma, glioblastoma and breast cancers. This is not surprising as a large body of work has shown the importance of STAT3 in embryonic stem cell differentiation in cooperation with other central regulators of pluripotency, including Oct4 and Sox family transcription factors as well as LIF [57]. Importantly, it there is an inverse relationship between AR expression and/or activity and the CSC phenotype in PCa cells [58,59] and downregulation of AR increases STAT3 signaling which is required for CSC maintenance in Du145 and TRAMP C2 PCa cells. In human prostate tumor tissues, elevated cancer stem-like cell markers coincide with those cells exhibiting high STAT3 activity and low AR expression [60]. Moreover, treatment of Du145 PCa that are positive for aldehyde dehydrogenase (ALDH), a marker of CSCs which express high levels of phosphorylated STAT3 with the potent and specific STAT3 inhibitor galiellalactone, reduces frequency of ALDH+ cells and induces apoptosis of Du145-ALDH+ cells [61]. Increased STAT3 signaling has also been observed in breast cancer ALDH+ stem cell populations and targeted inhibition of STAT3 reduces the tumorogenic capacity of this stem population [62]. Moreover, targeting STAT3 transcriptional activity using parthenolide, induces cell death in tumor initiating cells isolated from a number of PCa cell lines and prevents their growth *in vivo* [63]. 

The requirement for STAT3 in CSC maintenance in PCa is intrinsically linked to its role as a critical component of IL-6 signaling. The importance of the IL-6/STAT3 axis has been linked to supporting CSC populations in a variety of cancers, including hepatocellular [64], breast [65], head and neck cancers and glioblastoma [66,67]. In PCa, STAT3 activation associated with decreased AR expression is mediated through increased production of IL-6 and treating mice with soluble IL-6 receptor fusion protein significantly reduces CSC number and xenograft tumor growth [60]. Moreover, stem-like cells from patients with advanced PCa secrete high levels of IL-6 compared to normal prostate stem cells, and these cells express high levels of the IL-6 receptor and pSTAT3. In this study, they showed that inhibition of either IL-6 signaling using neutralizing antibody or a STAT3 inhibitor prevented the clonogenic potential of CSCs isolated from patients with high grade disease [68]. Moreover, IL-6/STAT3 signaling downstream of reactive oxygen species generation was found to be required for PCa spheroid formation [69]. 

Interestingly, this requirement for IL-6 signaling, in PCa CSCs may underlie the observation that there is significant overlap or fluctuation between a CSC and EMT-like phenotype in may PCa cell lines. Indeed, many reports in PCa as well as other cancers have shown a correlation between expression of EMT and CSC markers within the same cells. For example, after androgen deprivation, both EMT and CSC populations increase in mouse prostates and PCa cells [49] and PCa cells induced to an EMT phenotype, or CSCs isolated from PCa cell lines, strongly upregulate transcription factors expressed by CSCs or markers of EMT, respectively, and are highly tumorigenic in mice [70,71]. IL-6/STAT3 signaling may be a bridge between these phenotypes, as it has been identified as a driver of EMT in PCa that requires STAT3 [72]. Importantly however, new evidence suggests that IL-6 is not the only factor that can drive STAT3 dependent EMT in PCa. For example, CCL2-dependent STAT3 activation leads to EMT and inhibiting CCL2 prevents PCa cell line migration and invasion and *in vivo* xenograft growth better than AR targeting alone. Interestingly this mechanism occurs in cells with siRNA inhibition of the AR, further underscoring an inverse relationship between AR activity and the CSC/EMT phenotype [73]. In addition, ROS induction by EGF stimulation of PCa cells leads to transcriptional regulation of EMT via the E-Cadherin repressor Twist, which requires the phosphorylation of STAT3 and its subsequent activation of hypoxia inducible factor (HIF)1α [74]. TGF-β1 can also stimulate STAT3 phosphorylation and HIF-1α expression in PCa, leading to STAT3 and HIF-1α mediated Twist expression and increased invasiveness [75]. 

## 6. STAT3 and the Tumor Microenvironment in PCa

Despite the numerous cell intrinsic pathways that endow tumor cells with their remarkable propensity for unrestricted growth, survival and dissemination, the interaction of cancer with their host and the microenvironment tumors create for themselves play equally important roles in the progression of disease. This is of course true for PCa, and newly emerging roles of the stromal cells, immune cells and secreted factors that mediate the interactions between these cell types and the tumor in the pre-metastatic and metastatic niches are being uncovered at a rapid rate.

### 6.1. STAT3 in Angiogenesis

Tumor mediated angiogenesis is a hallmark of solid tumors [76]; they require the formation of new blood vessels to supply oxygen and nutrients that support their growth and survival. Vascular Endothelial Growth Factor (VEGF) is the most important inducer of tumor mediated angiogenesis [77,78] and STAT3 is a direct transcriptional activator of VEGF [79]. It is no surprise therefore, that inhibition of STAT3 reduces angiogenesis by reducing VEGF expression and therefore VEGF receptor activity in multiple models of cancer. Reciprocally, in breast, skin, pancreatic, cervical, head and neck carcinoma and prostate cancer cell lines expression of constitutively active STAT3 up-regulates VEGF expression and tumor angiogenesis [80,81,82]. Moreover, in PCa, the intersection of STAT3 and the AR also has important implications for VEGF expression, as there are AR binding sites in the promoter of VEGF, further controlling its transcription [83]. The expression of STAT3 also correlates with another highly potent angiogenic factor called basic-Fibroblast Growth Factor (bFGF) both tumor-derived myeloid cell lines, lung cancer cell lines as well as lung cancer patient samples. This correlation was proven functionally when experiments knocking down STAT3 reduced expression of bFGF [84,85]. The importance of STAT3 as a central signaling node in PCa downstream of bFGF and VEGF is further underscored by recent work showing that delivery of endostatin, a non-specific inhibitor of FGF and VEGF angiogenesis and STAT3 siRNA using an attenuated *Salmonella* vector inhibited PCa xenograft growth *in vivo* [86]. Interestingly, VEGF is also a target gene of the AR and its expression is dependent on STAT3 activity. Like in PCa, VEGF expression is important for hepatocellular carcinoma progression, which is often associated with hepatitis C viral infection, and HCV infection induces STAT3 and AR dependent VEGF expression [87].

As mentioned above, STAT3 also activates transcription of HIF-1α, which in itself is a transcriptional activator of VEGF, and STAT3 and HIF-1α cooperatively bind to the VEGF promoter to induce VEGF transcription [14]. Inhibition of the HIF-1α/STAT3/VEGF pathway using multiple plant derived agents and siRNA, have shown reduction of PCa tumor cell growth both *in vitro* and *in vivo* [88,89,90,91,92]. Moreover, combination therapy using specific inhibitors of HIF-1α and STAT3 greatly reduce growth of Du145 and TRAMP C2 PCa cell lines and xenografts [93]. Interestingly, a feed forward loop seems to exist in PCa between STAT3, oncogenic signaling and HIF-1α mediated angiogenesis; as STAT3 can directly inhibit p53 and p53 acts as an inhibitor of HIF-1α, it may be that in PCa where STAT3 is constitutively active there is further downstream activation of HIF-1α and VEGF. This idea is supported by work showing that overexpression of HDM2, an oncogene that supresses p53 in cancer cells, in LNCaP PCa cell lines increases pSTAT3, HIF-1α and VEGF expression [94].

### 6.2. STAT3 in the Stromal and Bone Compartments

Importantly, STAT3 signaling is not confined to regulating tumor cell intrinsic pathways that control the microenvironment; it is also a key player in the development of “reactive” or inflammatory stromal cells that have been shown to promote PCa tumor aggressiveness [95,96]. For example, activation of the canonical WNT/β-catenin signaling cascade in PCa stromal cells by fibroblast growth factor (FGF) activates STAT3 and promotes PCa tumor progression *in vivo* [97] and loss of TGFβ mediated suppression of Wnt3a via STAT3 in stromal cells promotes LNCaP cell proliferation and tumorogenesis *in vitro* and *in vivo* [98]. In addition, secreted heat shock proteins can also promote stromal reactivity in PCa. Secreted Hsp90 from PCa cell lines rapidly activates STAT3 in prostate fibroblast cell lines, leading to the activation of the pro-inflammatory NF-κβ pathway and secretion of inflammatory mediators known to augment PCa tumor survival and proliferation, such as IL-6 and IL-8 [99]. The activation of STAT3 in the stromal compartment is not limited to PCa; as examples, feed forward loops exist in breast [100] and ocular cancers [101] that induce the activation of STAT3 and subsequent secretion of CCL2 by cancer associated fibroblasts (CAFs) which promotes tumor cell survival and proliferation. Reciprocally, secreted factors, including FGF, EGF, HGF, chemokines and cytokines, from stromal cells have paracrine effects which activate STAT3 in tumor cells. Mesenchymal stem cells from hypoxic stroma secrete VEGF and the chemokine CCL21 to promote the STAT3 dependent activation of PI3K/AKT/NF-κβ and migration of PC3 PCa cell lines and xenografts [102]. Moreover, secretion of inflammatory cytokines, such as TNFα from cancer associated fibroblasts drives EMT in tumor cells through NF-κβ and AKT mediated stabilization of the E Cadherin repressor, Snail [103]. Since STAT3 is an important signaling component in both of these pathways in PCa, it is likely that it is also activated downstream of paracrine TNFα arising from the stromal compartment to promote PCa progression. 

While primary prostate tumors are supported through interactions with fibroblastic stromal cells, tumor cells that metastasize modulate hematopoietic stroma in the bone to establish themselves within that niche. Over 80% of men that die from CRPC have tumors that have metastasized to the bone. These bone lesions are primarily osteoblastic and a variety of bone and cancer derived growth factors and chemokines play important roles in tumor establishment and bone destruction in this metastatic niche [104]. One signaling pathway essential for normal bone metabolism and the pathogenesis of PCa bone metastases is the receptor activator of NF-κβ (RANK) pathway [105]. Interactions between RANK with its ligand in the bone compartment stimulates osteoclast differentiation and this pathway is also functional in PCa cells; Du145 and PC3 PCa cells expressing RANK respond to RANKL, increasing migration and invasive properties [106,107,108,109]. RANK signaling stimulates a variety of intracellular signaling cascades, including NF-κβ and AKT, in which STAT3 signals [105]. The importance of STAT3 in promoting bone metastasis in PCa is highlighted by the fact that that conditioned medium from PC3 PCa cells induces the production of chemokines and cytokines from osteoblasts that promote osteoclast generation and that STAT3 in osteoblasts is detectable in bone biopsies from patients with osteolytic metastases [110]. In addition, the canonical IL-6/STAT3 axis also plays crucial roles in the development of bone metastases for a number of solid tumors. IL-6 has multiple effects on RANK, TGFβ and Wnt signal cascades and the control of these pathways by IL-6 results in degradation of bone that facilitates cancer metastasis to this site [104]. While radiographically PCa causes osteoblastic lesions in the bone, it has been suggested that the presence of osteolytic lesions in addition lead to bone weakness [111] and interestingly, IL-6 mediates the formation of hematopoietic stem cell derived osteolytic lesions in a bone model of PCa [112]. 

### 6.3. STAT3 Mediated Immune-Suppression

An essential component of the tumor microenvironment that dictates cancer progression is the immune system. Indeed, evading detection and clearance by innate and adaptive immune cells is a hallmark of cancer [113] and STAT3 plays equally important roles in regulating leukocyte cell function as it does in tumor cells, putting it at the crossroads of tumor immune evasion. Many studies have shown persistent activation of STAT3 in myeloid cells and T cells at primary tumor sites, leading to an immunosuppressive environment which allows for tumor angiogenesis, growth and metastasis [114] More specifically, STAT3 mediated immunosuppression has been linked to its function downstream of cytokines and T cell inhibitory or “checkpoint” molecules, such as IL-27, PDL-1 and HVEM that promote the differentiation of regulatory T cell subsets [115,116,117,118]. In PCa, use of the histone deacetylase inhibitor entinostat inhibits the induction of the T regulatory cell transcription factor FOXP3 in a STAT3 dependent manner, resulting in decreased survival of CRPC tumors [119]. STAT3 also plays an important role in innate immune cells targeted by PCa cells. For example, hormone resistant TRAMPC2 xenograft tumors recruit myeloid derived suppressor cells (MDSCs), a cell type well known for their association with aggressive solid tumors, in an IL-6/STAT3 dependent manner, which promotes their growth *in vivo* [120]. Like MDSCs, tumor associated macrophages (TAMs) also can play substantial roles in preventing anti-tumor responses and macrophages co-cultured with prostate epithelial cells may be induced to this phenotype, producing the chemokine CCL4 in a STAT3 dependent manner which induces spontaneous prostate tumorogenesis in normal prostate cells associated with the downregulation of p53/PTEN as well as the induction of EMT [73,121]. CCL2-STAT3 activation also occurs in macrophages exposed to anti-androgens like Enzalutamide and Casodex, enhancing the migratory capacity of the macrophage and subsequent invasiveness of PCa cells. Reciprocally, the CCL2-STAT3 axis also is activated directly in PCa cells downstream of AR signaling and combination therapy targeting the AR and the CCL2/CCR2/STAT3 pathway effectively prevents EMT in PCa cells *in vitro* and suppresses xenograft growth *in vivo* [73,122]. The activation of STAT3 signaling pathways in PCa cells not only affects their interactions with macrophages and T cells, but B cells as well. Activation of IKKβ and STAT3 are activated in CRPC and that this signaling is required for the CXCL13 mediated recruitment of immunosuppressive B cells to the tumor microenvironment [123]. Importantly, as STAT3 signaling is central to transduction of signals from both IL-10 and IL-6 family cytokines as well as IFN, all key factors that shape the outcome of antigen presentation and effector T cell responses as well as PCa cell survival, it most certainly is a key driver of immunosuppression and tumor survival not just in PCa but for all malignancies. 

### 6.4. STAT3 as a Therapeutic Target for PCa

Considering the wide array of signaling pathways that promote progression of CRPC that require STAT3, targeting this molecule for therapeutic benefit has been an intense area of investigation. This is true not only for PCa, but many other cancers as well. In Table 1, the extensive list of natural compounds, JAK kinase inhibitors, small molecules and cytokine pathway antibody therapies that can inhibit STAT3 function and their experimental effects on PCa are presented. Importantly, while all of these compounds are known to inhibit IL-6, JAK and/or STAT3 signaling, some of the listed effects on PCa cell lines and *in vivo* models were not necessarily associated with inhibition of STAT3 itself. While many of these agents have anti-proliferative effects on PCa *in vitro* or in *in vivo* xenograft models in immunocompromised animals, few have gone on to show efficacy as single agents in patients. For example, the IL-6/JAK/STAT3 inhibitors siltuximab (CNTO 328) and ruxolitinib (INCB-018242) failed Phase II clinical trials due to a complete lack of PSA*50* response [124,125]. The failure of these IL-6 inhibitors as monotherapies may most likely be explained by the wide variety of other secreted factors that are known to activate STAT3 in solid tumors, including many of those mentioned above like VEGF, bFGF, EGF, chemokines (CCL2, 21, *etc*.) and cytokines in the IL-6 family. Importantly, in the studies aforementioned, most inhibition of PCa growth, migration, invasiveness or metastasis in *in vitro* or *in vivo* models was achieved using STAT3 specific inhibitors rather than inhibition of IL-6. However, combination therapy using IL-6 targeting with chemotherapy may prove more effective, as a recent phase I trial with siltuximab and docetaxel in combination showed robust PSA decline in the patients [126]. While these results do not corroborate findings from a previous phase II study in CRPC patients combining silutixmab with mitxantrone, which showed no difference in progression free survival [127], this may be due to an inappropriate choice of endpoint in this study [128]. 

By contrast however, targeting Hsp27 has shown promise in PCa clinical trials in combination with prednisone in patients with metastatic CRPC; up to 71% of patients were progression free at 12 weeks and 50% showed 50% reduction in PSA when treated with the Hsp27 antisense OGX-427 and prednisone [129]. While these effects are most certainly not limited to Hsp27 effects on STAT3, preclinical studies using OGX-427 have shown that there is significant reduction of STAT3 activity in LNCaP with this treatment and that the cytoprotection afforded to LNCaP cells by Hsp27 overexpression requires STAT3 [130]. The heterogeneous nature of PCa tumors combined with the multifaceted roles of STAT3 in survival and activation pathways in tumor cells, stromal cells and immune cells may answer why there is such diversity in effects of STAT3 inhibitors in patient responses; certainly future explorations will entail targeted combination therapy of STAT3 inhibition with other key pathways identified in individual patient tumors that have actionable targets.

**Table 1:** List of different drugs targeting STAT3 signaling pathway in cancers.

Drug	Mechanism	Effects on PCa	Ref.
*Inhibitor class: Natural Products*			
Curcumin*	Dietary spice that has been shown to inhibit JAK1, JAK2 and therefore STAT3 tyrosine phosphorylation.	Inhibits AR expression	[131,132,133]
Guggulsterone	Stimulates tyrosine phosphatases responsible for de-phosphorylation of STAT3.	Causes apoptosis in AR- PC3 cells through STAT3 inhibition.	[134,135,136]
Capsaicin*	Inhibits JAK1 mediated STAT3 phosphorylation but also induces tyrosine phosphatases.	Induces apoptosis *in vitro* and *in vivo*.	[137,138]
Celastrol	Inhibits IL6 induced JAK2 phosphorylation of STAT3.	Inhibits the TMPRSS-ERG fusion.	[139,140]
Caffeic acid (CA) CAPE CADPE	Caffeic acid and its derivatives all inhibit STAT3 phosphorylation by blocking JAK2 activity along with other tyrosine kinases like Src.	Anti-proliferative and anti-androgenic activity.	[141,142,143]
Curcubitacin B E F	Chinese medicine family ranging from Curcubitacin A to T. Curcubitacin B has been studied the most and it prevents STAT3 phosphorylation by inhibiting JAK2.	Curcubitacin E disrupts cytoskeleton in PCa cell lines.	[144,145]
Cryptotanshinone*	Binds to SH2 domain of STAT3 and prevents dimerization.	Inhibits STAT3 in PCa cell lines and suppresses AR activity,	[146,147]
3,3′-diindolyl-methane*	DIM has various anti-cancer properties. It inhibits JAK2 function. It is also important to note that DIM has anti-androgen activity.	Heavily tested in PCa, affects AR activity, metastasis, epigenetics, Currently in Phase II clinical trials for PCa.	[148,149,150,151,152,153]
Emodin	Pugrative resin extracted from rhubarb. Has various pharmacological activities including inhibition of JAK2.	Inhibits PI3K pathway and AR activity in PCa cell lines.	[154,155,156]
Paclitaxel*	Inhibit STAT3 phosphorylation and STAT3 interaction with tubulin.	Has been tested in many Clinical Trials for CRPC and metastatic PCa.	[157,158]
Evodiamine	Suppresses pY-STAT3 by inducing expression of tyrosine phosphatase SHP-1.	Causes apoptosis in various PCa cell lines.	[159,160,161]
Indirubin	Block VEGFR induced phosphorylation of JAK2 and consequently STAT3.	Induces apoptosis and reduces angiogenesis in PCa cell lines via STAT3 inhibition.	[92,162,163]
*Inhibitor class: STAT3 Small Molecule Inhibitors*			
S31-1757	Binds to the SH2 domain of STAT3. The inhibitor binds to Arg-609 and Lys-591; both sites are essential in recognition and binding to the pTyr-705 residue of STAT3 (dimerization) as well as the pTyr-904 for binding to the gp-130 subunit of the IL-6 receptor as well as other receptors like EGFR.	Not yet tested in PCa	[164]
Sttatic	Small-molecule that directly binds to the SH2 domain of STAT3 preventing the interaction with the phosphor-tyrosine motif of the neighbouring STAT3.	Not yet tested in PCa	[165]
STA-21	Also known as Ochromycinone was discovered through a virtual database screen in silico and was shown to inhibit STAT3 SH2 and phosphor-Tyr interaction.	Tested against some PCa cell lines where it reduces growth through pY-STAT3.	[166,167]
S31-201	Benzoic acid that was also discovered through *in silico* screen also inhibits dimerization of STAT3 through the SH2 domain.	Not yet tested in PCa	[168]
BP-1-102	Software designed analog of S31-201 whose structural differences allow it to interact with all 3 strutural sub-pockets in the SH2 domain of STAT3 causing a more potent inhibition of the dimerization.	Not yet tested in PCa	[169]
LLL12	Binds directly to Tyr705 of STAT3 to prevent phosphorylation and subsequent dimerization.	Not yet tested in PCa	[170]
*Inhibitor class: Kinase inhibitors*			
SAR302503*	Orally available inhibitor of Janus Kinase 2 (JAK-2).	Reduces tumor growth *in vivo* through suppression of STAT3.	[171,172]
LS104	A non-ATP-competitive small molecule inhibitor of JAK-2. This attribute of LS104 allows it to be used in combination with an ATP-competitive inhibitor for a synergistic effect.	Not yet tested in PCa	[173]
Atiprimod	Cationic amphiphilic compound that blocks transcription of IL-6 by inhibiting the NFκβ pathway as well as inhibits the phosphorylation of STAT3 at Tyr705 through a separate mechanism.	In clinical trials for Neuroendocrine Carcinoma. Could have implications in PCa.	[174,175,176,177]
Ruxolitinib (INCB-018242)*	Orally available a JAK1 and JAK2 inhibitor.	Failed Clinical Trials in metastatic PCa.	[124,178]
Lestaurtinib (CEP-701)	Inhibitor of a few tyrosine kinases including JAK2. It is structurally similar to staurosporine.	Suppresses AR activity.	[179,180]
Tofacitinib	Primarily a JAK3 inhibitor, but has some activity against JAK1 and therefore reduces pY-STAT3.	Not yet tested in PCa.	[181]
CYT387*	ATP competitive JAK1 and JAK2 inhibitor.	Not yet tested in PCa.	[182]
Pacritinib*	Orally available inhibitor for JAK2.	Not yet tested in PCa.	[183]
Sorafenib* SC-1 SC-49	Sorafenib and its derivatives are tyrosine kinase inhibitors that affect multiple kinases, including JAK2. They reduce pY-STAT3.	Currently in many clinical trials for metastatic PCa.	[184,185]
AZD1480*	ATP-competitive JAK2 inhibitor.	Suppresses growth of PCa cell lines.	[186,187]
Auranofin	A gold compound that inhibits STAT3 phosphporylation through JAK1 and also inhibits NFκβ activity.	Not yet tested in PCa.	[188,189]
AG-490	Known as Tyrophostin B42 is a potent inhibitor of Janus Kinase 2 (JAK2).	Induces apoptosis by supressing STAT3 activity.	[190,191,192]
XZH-5	Inhibits Tyr705 phosphorylation and dimerization of STAT3 and possibly targets one of the tyrosine kinases responsible for this: mechanism is unknown.	Not yet tested in PCa.	[92,193,194]
FLLL32	Derived from Curcumin, this compound prevents phosphorylation of STAT3 by inhibiting JAK2.	Not yet tested in PCa.	[195]
BMS-911543	Orally available small molecule JAK2 inhibitor. Active against V617F JAK2 mutants.	Not yet tested in PCa.	[196,197]
AC-430	Small molecule JAK2 inhibitor, also active against the V617F mutant.	Not yet tested in PCa.	[1]
CEP-33779	Small molecule JAK2 inhibitor.	Not yet tested in PCa.	[198,199,200]
R723	Small molecule JAK2 inhibitor, also active against the V617F mutant.	Not yet tested in PCa.	[201,202]
*Inhibitor class: IL-6 Antibodies/Inhibitors*			
Sant7	superantagonist of the IL-6 receptor capable of blocking all IL-6 receptor activity and therefore the activity of one of its major downstream transcription factors: STAT3.	Sensitizes PCa cell lines to cytotoxic therapy by inhibiting IL6/JAK/STAT3 pathway.	[203,204]
Tocilizumab	Humanized monoclonal antibody against the human IL-6 receptor. Works against both soluble and membrane bound IL-6R.	Not yet tested in PCa.	[205]
Siltuximab (CNTO 328)*	Chimeric murine-human monoclonal IL-6 antibody.	Failed Phase II clinical Trials in CRPC.	[127,206]
*Inhibitor class: DNA or RNA targeting*			
Platinum Compounds CPA-1 CPA-7 IS3-295 Carboplatin Oxaliplatin Satraplatin	Complexes like CPA-1, CPA-7 and IS3-295 disrupt the STAT3 interaction with DNA in breast, prostate, lung and skin cancers. The exact site where these complexes bind to STAT3 is unknown.	Platinum Compounds such as carboplatin, oxaliplatin or satraplatin have been used as chemotherapy agents in CRPC in clinics.	[207,208,209]
Double-Stranded Oligodeoxynucleotides decoys*	DNA sequences that are the same as GAS or ISRE elements and would bind to the STAT3 dimers in place of the actual sequence in the genome.	Not yet tested in PCa	[210,211]
G-rich oligodeoxynucleotides (G quartets)	Very specific K^+^-dependent four-stranded DNA structures that occupy sites within the STAT3 SH2 domains. The selection method for these G quartets can effectively be used to block any interaction in the cell.	Supress growth in PCa cell lines through STAT3 inhibition.	[212,213]
siRNA for STAT3	Through formation of double stranded mRNA, siRNA can degrade mRNA for specific proteins using the DICER enzyme.	Repeatedly shown to reduce STAT3 activity *in vitro* and *in vivo*.	[214,215,216]

*: Drugs are in clinical trials for different cancers.

## 7. Conclusions

A wide body of work spanning multiple solid tumor types, including PCa, have shown an indespensible and multifaceted role for STAT3 signaling in promoting tumor progression. Importantly, STAT3 has pivotal roles in re-activation of the AR, an essential step in the development of mCRPC. This can occur via STAT3 cooperation with other highly relevant oncogenic signaling cascades such as PTEN/PI3K/AKT and the integration of signals by STAT3 received from multiple growth factors, Src kinases and serine/threonine kinases that themselves have known roles in the progression of PCa. The diversity of signaling pathways in PCa tumor cells in which STAT3 acts as a major signaling node translates into it being a driving force in not only tumor proliferation and survival, but also in determining cell phenotype, behavior and interaction with stromal cells and the immune system. It is not surprising therefore that therapeutic targeting of STAT3 is under intense investigation for the treatment of mCRPC. However, due to the promiscuous nature of STAT3 signaling in so many cell types, the future success of potential therapies may possibly entail cell-specific delivery of STAT3 targeting or co-targeting with other known drivers of PCa progression to obtain optimal results. This concept has been explored to enhance immune responses in hematopoietic cancers, whereby targeting STAT3 specifically in TLR9 expressing cancer cells can be achieved using CpG based delivery of silencing oligos results in growth inhibition of myeloma and myeloid leukemia [217,218]. Indeed this work also highlights that STAT3 targeting can also be a modality of immunotherapy, which will surely also be a critical feature of the future of mCRPC treatment [219]. Taken together, it is clear that STAT3 sits at the crossroads of multiple signalling pathways that are essential for the progression of PCa to advanced disease and uncovering specific mechanisms of action of STAT3 in PCa tumor cells, stromal cells and associated hematopoietic cells will bring the PCa community closer to exploring STAT3 as a therapeutic target.
